# Porous Polymer Structures with Tunable Mechanical Properties Using a Water Emulsion Ink

**DOI:** 10.3390/ma17051074

**Published:** 2024-02-26

**Authors:** Joshua Z. R. Dantzler, Sofia Gabriela Gomez, Stephanie Gonzalez, Diego Gonzalez, Alan O. Loera Martinez, Cory Marquez, Md Sahid Hassan, Saqlain Zaman, Alexis Lopez, Md Shahjahan Mahmud, Yirong Lin

**Affiliations:** 1 Department of Aerospace and Mechanical Engineering, The University of Texas at El Paso, El Paso, TX 79968, USA; jzdantzler@miners.utep.edu (J.Z.R.D.); sggomez@miners.utep.edu (S.G.G.); sgonzalez80@miners.utep.edu (S.G.); aoloera@miners.utep.edu (A.O.L.M.); mhassan2@miners.utep.edu (M.S.H.); szaman3@miners.utep.edu (S.Z.); alopez125@miners.utep.edu (A.L.); mmahmud4@miners.utep.edu (M.S.M.); 2 Department of Computer Science, The University of Texas at El Paso, El Paso, TX 79968, USA; dgonzalez14@miners.utep.edu; 3 Sandia National Laboratories, Albuquerque, NM 87185, USA; cmarquez10@miners.utep.edu

**Keywords:** polydimethylsiloxane (PDMS), foam, rheology, 3D printing, tunable mechanical properties

## Abstract

Recently, the manufacturing of porous polydimethylsiloxane (PDMS) with engineered porosity has gained considerable interest due to its tunable material properties and diverse applications. An innovative approach to control the porosity of PDMS is to use transient liquid phase water to improve its mechanical properties, which has been explored in this work. Adjusting the ratios of deionized water to the PDMS precursor during blending and subsequent curing processes allows for controlled porosity, yielding water emulsion foam with tailored properties. The PDMS-to-water weight ratios were engineered ranging from 100:0 to 10:90, with the 65:35 specimen exhibiting the best mechanical properties with a Young’s Modulus of 1.17 MPa, energy absorption of 0.33 MPa, and compressive strength of 3.50 MPa. This led to a porous sample exhibiting a 31.46% increase in the modulus of elasticity over a bulk PDMS sample. Dowsil SE 1700 was then added, improving the storage capabilities of the precursor. The optimal storage temperature was probed, with −60 °C resulting in great pore stability throughout a three-week duration. The possibility of using these water emulsion foams for paste extrusion additive manufacturing (AM) was also analyzed by implementing a rheological modifier, fumed silica. Fumed silica’s impact on viscosity was examined, revealing that 9 wt% of silica demonstrates optimal rheological behaviors for AM, bearing a viscosity of 10,290 Pa·s while demonstrating shear-thinning and thixotropic behavior. This study suggests that water can be used as pore-formers for PDMS in conjunction with AM to produce engineered materials and structures for aerospace, medical, and defense industries as sensors, microfluidic devices, and lightweight structures.

## 1. Introduction

Porous materials are a major focus of material science, providing versatility for many applications. One of the earliest known applications of porous materials was by the ancient Egyptians, who used charcoal to aid indigestion [1]. Later, European scientist Carl Scheele, studied the adsorption of gases by charcoal. In the twentieth century, higher gas adsorption materials were made possible by porous polymer networks. Porous polymers consist of polymeric materials with various pore sizes, classified as microporous, mesoporous, and macroporous with pore diameters of less than 2 nm, 2–50 nm, and greater than 50 nm, respectively [2]. Porous polymers find their applications in diverse fields, including energy storage and biomedical use. Liu et al., demonstrated porous polytetrafluoroethylene (PTFE) and Nafion composites for fuel cell applications. The PTFE-Nafion composite worked successfully, with the fuel cell performance increasing with pore size [3]. Liu et al., noted that after a 180 h stability test at 500 mA/cm^2^, there was no voltage drop in the cells. In biomedical applications, the presence of porous structures can mimic bone tissue, making them a strong candidate for lightweight load-bearing structures. Paljevac et al., demonstrated the fabrication of macroporous poly(methyl methacrylate) (PMMA) through hard spheres and high internal phase emulsion where human osteoblast cells were grown on these materials [4]. Kim et al., explored this phenomenon further by using porous poly(lactic-co-glycolic acid) (PLGA) as a scaffold to introduce the bio-compatible ceramic hydroxyapatite (HA) by coating the polymer-ceramic in apatite to increase the osteogenic potential [5]. It was found that, when exposed to simulated body fluid, the apatite growth was substantial, proving the capability of the PLGA/HA scaffolds for bone tissue engineering. Porous polymers are versatile in their use and find an application in the purification of water and liquid repellence due to their hydrophobic nature. Das et al., engineered a catalyst-free reaction to integrate graphene oxide into polymeric materials with outstanding antifouling properties, tunable mechanical properties, versatility in shape formation, and excellent hydrophobic coatings for any material, flexible or rigid [6] The existence of an excellent water-repellant material, as well as its moldability, leave many potential uses, such as protein detection and crystallization, tissue engineering, and drug sensing. Moreover, the antifouling property resisted bending, creasing, tweeting, and erosion of the top surface. The wettability may be altered from nonadhesive to adhesive superhydrophobicity, with the ability to develop reversible aqueous patterns on the polymeric coating, opening more avenues of applications, such as water harvesting and microfluidic devices [7].

Among porous polymers, polydimethylsiloxane (PDMS) is of interest in this scope because it is a biocompatible polymer commonly used in implants and flexible electronics in the medical industry [8,9,10,11]. PDMS is highly desirable due to its excellent optical, mechanical, thermal, and electrical properties [12]. PDMS can be optically transparent, which enables the use of microfluidics capable of fluid manipulation on a small scale [13]. The mechanical properties aid medical use due to its great elasticity. Kang et al., produced a flexible pressure sensor from PDMS, opening new opportunities for wearable sensors [14]. Due to its low thermal conductivity and electrical insulation, PDMS is commonly used in encapsulated electronics. To enable the formation of pores, PDMS must be combined with pore-formers or expanding agents. These are liquid additives that expand and create pores. Mikolaszek et al., investigated the effects of various pore-formers for the application of controlled drug release [15]. Here, they infiltrated PDMS with silicone oil (SO), polyoxyethylene glycol (PEG), and propylene glycol (PG). It was found that PEG increased the flux of the active substance indomethacin.

Reporting of traditionally manufactured PDMS in the literature is quite commonplace. Historically, it is fabricated conventionally through molds. With the improvement in technology, PDMS may be fabricated through additive manufacturing (AM) with designed application-driven structures. AM is a fabrication technique in which material is manufactured on a layer-by-layer basis, enabling unique designs and properties [16]. One form of AM is material extrusion, where the material is extruded through a small orifice through means of pressure [17]. Direct ink write (DIW) printing, which is under material extrusion, is a technique that consists of layer-by-layer deposition of material through a nozzle with a pressure-driven mechanism onto a substrate [18]. DIW printing provides many advantages, such as enabling combinations of different formulations into complex structures and modifications of the extrusion system by incorporating microfluidic printheads, allowing different materials in a single pass [19]. Some limitations of DIW printing include additional post-process treatments, weak mechanical properties, and specific rheological requirements of inks [20]. Ozbolat et al., report that PDMS manufactured through material extrusion boasts higher mechanical properties in comparison to cast samples [21]. Alternatively, Femmer et al., achieved a stellar resolution of 100 μm by prototyping PDMS through digital light processing [22]. This enables the rapid printing of microfluidic chips and various membrane devices. However, 3D printing of pore-former-enabled porous PDMS is rarely reported in the literature. Chen et al., printed liquid PDMS filled with salts and silicone oil as the pore-former [23]. The print was then immersed in alcohol for 5 h, followed by water immersion at 90 °C for 10 h. Then, the pore-formers were removed, resulting in the creation of a PDMS sponge with macro and micropores. Woo et al., set out to investigate the structure–mechanical relationships of 3D printed porous PDMS, also using material extrusion 3D printing [24]. Dibutyl phthalate (DBP) was added to the PDMS ink as the pore-former. The printed structure was thermally cured for 2 h at 100 °C. To create pores, DBP was removed by an ethanol bath at 60 °C for 1 h. Since the structural-mechanical properties of porous PDMS have only been studied in tension, it is important to also investigate the compressive properties of porous PDMS.

This study focuses on creating a water emulsion foam precursor for DIW 3D printing, optimizing emulsification, and analyzing storage conditions for porous PDMS precursors. The influence of tunable viscosity on porosity percentages and pore size distribution was also studied with the integration of fumed silica. Most interestingly, the reduction in post-processing has been shown in this work, in contrast to other works. Post-processing, such as solvent leeching or thermal etching, is usually needed to remove the pore-formers. Rather, in this study, curing and simultaneous evaporation of liquid water from the printed structure is demonstrated, resulting in a fully cross-linked and porous water emulsion foam. Additionally, compression testing reveals the foams’ Young’s Modulus, ultimate compressive strength, shape retention, and energy absorption properties. This work demonstrates the facile tunability of PDMS foams in the synthesis stage. This can result in rigid structures, which benefit microfluidic applications due to the need for enhanced structural integrity [25]. Conversely, this novel method can also result in flexible structures, which are great for wearable sensors as the human body is in perpetual movement [26]. Work shown in this project will benefit many applications, such as aerospace, defense, and medical, in which high strains are exerted and mitigation of shock is needed while maintaining a relatively low density within the specimens.

## 2. Materials and Methods

### 2.1. Materials

Sylgard 184 and its analogous curing agent or catalyst were ordered as a kit and (Dow Corning, Torrance, CA, USA) were used as the PDMS precursor in investigations with varying amounts of distilled water. Dowsil SE 1700 and its corresponding catalyst (Dow Corning Torrance, CA, USA) were included in the PDMS precursor studies that investigated higher viscous water emulsion foam inks. Fumed silica particles of 40 nm (AEROSIL OX50, EVONIK INDUSTRIES, Parsippany, NJ, USA), with a specific surface area of 35–65 m^2^/g, were used to modify the water emulsion foam ink rheology. FiberGlast 1153 was used as the mold release agent (FibreGlast, Brookeville, OH, USA) and was used to prevent the adhesion of water emulsion foams to respective molds.

### 2.2. Formulation of Water Emulsion Foams with Varying Water Percentages

**Figure 1:**
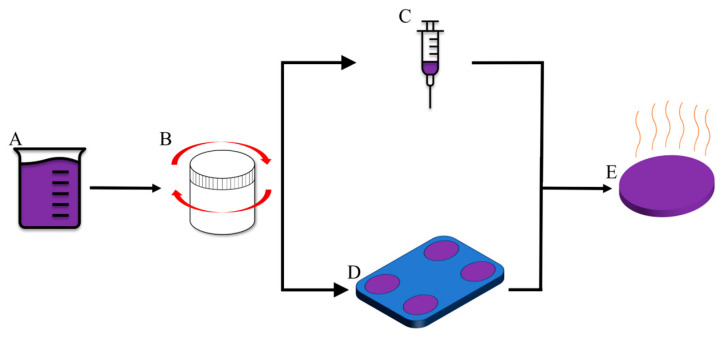
Foam Preparations, (**A**) PDMS synthesis, (**B**) centrifugal mixing, (**C**) DIW printing, (**D**) mold preparation, (**E**) thermal curing.

To prepare the water emulsion foam precursor, a planetary centrifugal mixer machine, as schematically shown in Figure 1, (Thinky Mixer ARE-310, Thinky Inc., Laguna Hills, CA, USA) was used to mix Sylgard 184 base and curing agent at the recommended 10:1 weight ratio. The base was added with distilled water to prepare seven different water emulsion compositions. Several different weight percentages were synthesized, containing PDMS-to-water ratios ranging from 90:10, 75:25, 65:35, 50:50, 35:65, 25:75, to 10:90. Each composition began with a mixing time of 4 min at 2000 rpm. However, with increasing water percentage, it became difficult to mix the hydrophobic and hydrophilic phases. Furthermore, mixing times were increased in increments of 4 min until a homogenous white water emulsion foam precursor was obtained with the absence of large water or air bubbles. The Sylgard 184 curing agent was implemented last to reduce the chances of early curing of PDMS due to amplified mixing durations. When the mixing process was completed, the water emulsion foam precursor was poured into an acrylonitrile butadiene styrene mold that was 3D printed using a fused filament fabrication 3D printer (CREALITY, Shenzhen, China). The dimensions for the mold were chosen based on ASTM D575 standards for compression specimens of rubber materials [27]. The water emulsion foam specimens were degassed in a vacuum desiccator for 90 min until no visible air bubbles were present. No surfactant was used to prevent chemical changes to the PDMS polymer structure. Despite this, fine emulsions were still fabricated. After degassing, the foams were thermally cured in a two-step process in a box oven (KSL-1500X, MTI CORPORATION, Richmond, CA, USA). Current literature was referenced before thermal post-processing to ensure that curing and pore creation did not degrade the material by staying well below the decomposition temperatures of Sylgard 184 and SE 1700: 400 °C and 430 °C, respectively [28,29]. The initial ramp rate used was 1 °C/min until the temperature reached 90 °C and dwelled for 120 min to cure the PDMS matrix. The next ramp was 1 °C/min until the temperature reached 120 °C and dwelled for 120 min to evaporate the water inside the pores.

### 2.3. Preparation of Water Emulsion Foams with Varying PDMS Viscosities

**Figure 2:**
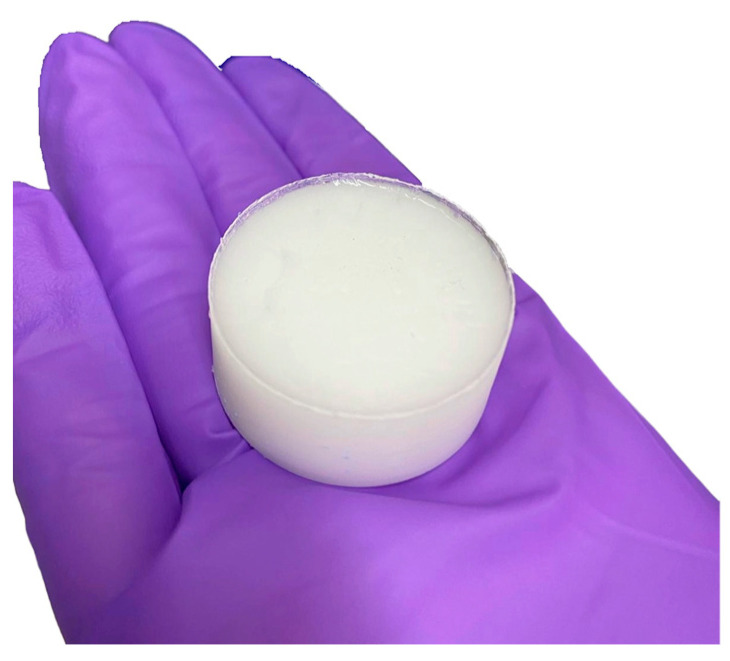
50:50 PDMS-to-water sample.

To observe the mechanical properties and porosity of the foams, specimens A, B, C, and Z (bulk sample) were prepared with PDMS-to-water ratios of 65:35, 35:65, 10:90, and 100:0, respectively. After mechanical testing, which is further elaborated on in Section 3.2, the PDMS-to-Water ratio is fixed at 65:35. With this fixed PDMS-to-Water ratio, viscosities were then analyzed by preparing samples with varying weight percentages of Sylgard 184 to Doswil SE 1700: 100:0, 90:10, 80:20, 70:30, and 60:40, specimens A, D, E, F, and G, respectively. A 50:50 weight ratio was unsuitable because its viscosity was too high, inhibiting the homogenous mixing with water to form the PDMS porous structures. While the viscosity of the PDMS was increasing, a constant 75:25 PDMS-to-water ratio was employed for consistency and observance of varying the single viscosity parameter. The bases of both Sylgard 184 and Dowsil 1700 are measured at the respective weight percentages and mixed for 4 min at 2000 rpm. Following the base mixture, water is integrated and mixed for 4 min at the same rotational speed. As previously stated, the mixing time may need to be increased at this step to achieve a homogenous mixture. Finally, the catalysts are mixed at 2000 rpm for 4 min. This is the last step to prevent premature curing through heat dissipated from the Thinky mixer due to high mixing times. The ink is dispensed, degassed, and cured in the same way as stated in Section 2.2. A characteristic 50:50 PDMS-to-water sample may be seen in Figure 2. Macroscopically, all specimens bear the same physical appearance as the characteristic sample and are indistinguishable from one another.

### 2.4. Preparation of Water Emulsion Ink for DIW

The formulation of water emulsion foam inks for material extrusion additive manufacturing must be tailored to improve shear thinning and shape retention. The PDMS precursor and water are first mixed in the Thinky mixer, as described in Section 2.2. After achieving a homogeneous solution of PDMS and water, 1 wt%, 3 wt%, and 9 wt% of fumed silica were added to the mixing cup and mixed at 2000 rpm for 20 min for Trials 2, 3, and 4. Weight percentages above 9 wt% were considered too viscous for printing and thus were excluded from the rheological study. The PDMS-water-silica mixture is then deposited into a 3 mL printing syringe, supplied by BioX (Bio X^TM^, CELLINK, San Diego, CA, USA) and is placed in a vacuum desiccator to remove trapped air bubbles. To avoid the significant movement of water-filled pores, the mixture is only placed in a vacuum for 30 min while in the syringe. The CAD file used for printing was a 1 × 1 in. square 2D lattice that was printed in a serpentine channel fashion. This CAD file was not created in software and was preloaded on the printer. Printing parameters were explored and involved varying printing needle diameter, printing speed, and air pressure to enable fluid flow. A total of four trial prints were executed using a 22-gauge needle, a printing speed of 2 mm/s, and an air pressure of 40 kPa.

### 2.5. Characterization

The water emulsion foam microstructure was analyzed using scanning electron microscopy (SEM) imaging using the Phenom ProX Desktop SEM (ThermoFisher Scientific, Waltham, MA, USA) with a beam intensity of 15 kV [30]. Sputtering was not performed as high-resolution images of the structured porosity were obtained. ImageJ software (ImageJ 1.53m, U.S. National Institutes of Health, Bethesda, MD, USA) was used to calculate the porosity of the foams after curing. Density calculations were taken before and after curing the water emulsion foams to determine relative density. An Amscope optical microscope ME520T (MICROSCOPE CENTRAL, Feasterville, PA, USA) was used to visualize the macroscopic characteristics of the polymer foams. The compressive properties were tested using the Instron 5578 load frame (Instron, Norwood, MA, USA) employed with a 10 kN load cell. Specimens were subjected to a strain rate of 12 mm/min until reaching 50% uniaxial compression. Water emulsion foam ink rheology was measured by a rotational rheometer DHR-2 (TA Instruments, New Castle, DE, USA). Shear thinning was examined by steady-state flow sweep tests with an increasing shear rate range from 0.01 to 100 s^−1^. Shape retention was characterized through oscillatory tests using a frequency of 1 Hz and a shear rate ranging from 0.1 to 1000 s^−1^.

## 3. Results and Discussion

### 3.1. Microstructure Characterization and Porosity

**Figure 3:**
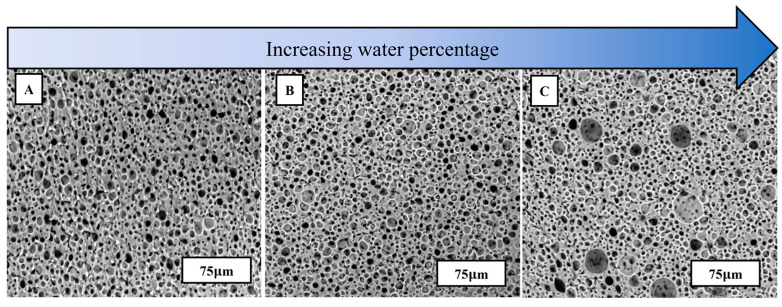
SEM images of water emulsion foams with varying PDMS-to-Water ratios, (**A**) specimen A, (**B**) specimen B, and (**C**) specimen C as described in Table 1.

SEM was used to capture the microstructure of porous PDMS after immediate cure, as shown in Figure 3. ImageJ software was used to capture the average diameter and porosity percentage of the water emulsion foams. The compositions of PDMS-to-water are 100:0, 65:35, 35:65, and 10:90 and their porosities and average diameters are listed in Table 1. Figure 3 shows the effects of increasing water content on pore size distribution. From left to right, the water content increases, therefore increasing the average porosity percentage. However, there is a slight decrease in porosity with PDMS-to-water ratios higher than the threshold of 35:65. The decrease in porosity can be attributed to water agglomerates from excessive amounts of water within a single specimen. The highest average porosity is achieved by specimen B, which resulted in a 55.5% porous PDMS structure.

### 3.2. Uniaxial Compression Testing of Water Emulsion Foams

**Figure 4:**
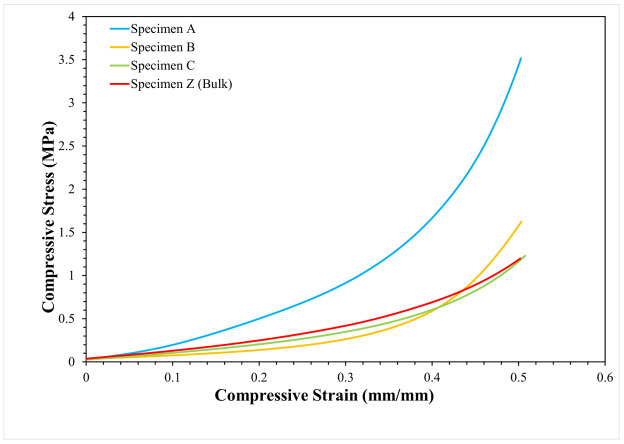
Stress-strain curves for specimens A, B, C, and bulk PDMS generated through compression testing.

Compression testing was conducted at a compressive strain of 12 mm/min according to ASTM D575 [27]. Table 2 shows the quantification of the modulus of elasticity, energy absorption, and compressive strength for the water emulsion foams tested. To save time yet show a clear pattern, specimens A, B, C, and bulk PDMS were tested as they host a breadth of PDMS-to-water ratios from the maximum amount to the lowest amount of PDMS. The stress-strain curves for specimens A, B, C, and bulk PDMS are shown in Figure 4. The compressive properties of bulk PDMS were investigated to comprehend the effects of pores in the PDMS matrix. The highest overall compressive strength, energy absorption, and modulus were achieved by specimen A. To recall, specimen A had the lowest porosity of the three specimens tested, at about 30%. Specimen A also exhibited the lowest average pore size distribution, with about 4.22 micron-sized pores on average. The microscopic characteristics of specimen A are attributed to its mechanical performance, and based on the results in Table 2, it outperforms all other specimens tested. Specimen A’s modulus was 193% and 60% higher than that of specimens B and C, respectively. The energy absorbed by specimen A was 200% higher than that of specimen B and 154% higher than specimen C. These results lie congruent with current data on mechanical testing of porous PDMS. During tensile testing, Woo et al., reported an 80% increase in ultimate tensile strength in a porous sample of PDMS compared to a non-porous sample [24]. This phenomenon can be attributed to the pore’s effect on the mechanical properties. The pores provide flexibility and durability, effectively cushioning the sample and increasing the mechanical properties. Later, Woo et al., implemented varying printing patterns during printing, changing the infill density and altering the porosity further. With a 90–90 print pattern, where PDMS is extruded in a grid-like fashion for each layer, Woo reported 75% infill having the highest modulus, ultimate tensile strength, and elongation at break when compared to 100% infill. However, there seems to be a lower limit to the benefit that porous structures provide. Woo et al., reported reduced mechanical properties of the 50% infill sample for the 90–90 orientation when compared to 100% infill. This occurrence draws a parallel conclusion to the data presented herein, where sample C (51.9% porosity) has reduced compressive strength compared to the bulk sample until the break and a lower modulus of elasticity. Specimen B underperforms compared to the bulk material until it experiences increased strain, where it has a sharp stress increase. This is due to the pores collapsing and effectively making the material denser.

### 3.3. Storage Life Assessment of Water Emulsion Foams

**Figure 5:**
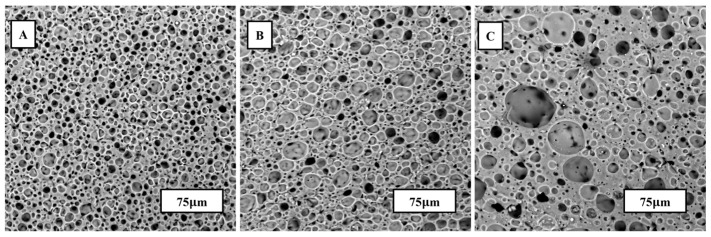
SEM images of specimen B (**A**) immediately after curing, (**B**) after 48 h (**C**) after 7 days of refrigerated storage.

**Figure 6:**
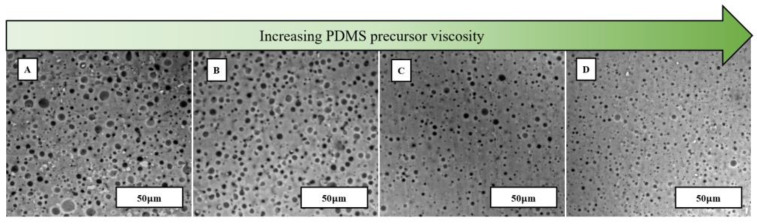
SEM images of water emulsion foams with varying Sylgard 184: SE 1700 ratios, (**A**) specimen D, (**B**) specimen E, (**C**) specimen F, and (**D**) specimen G, as described in Table 3.

**Figure 7:**
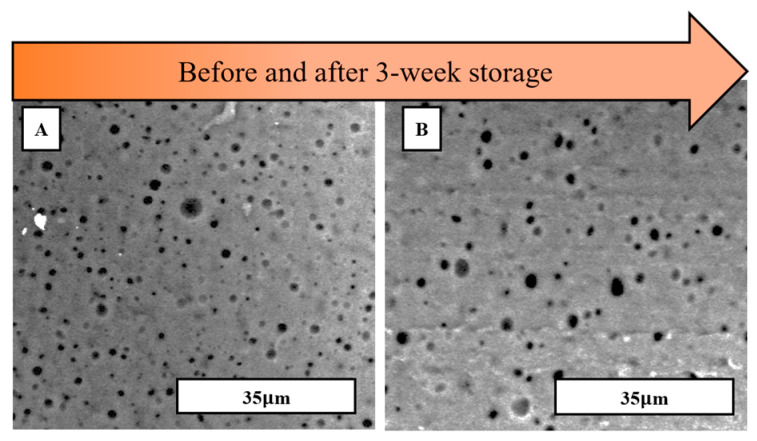
SEM images of water emulsion foams with optimized storage procedure for specimen A (**A**) before and (**B**) after a 3-week storage life in freezing temperatures.

The storage life of the water emulsion foams was investigated to observe the stability of the water-filled pores. Specimen A remained unchanged, and specimens D through G have increasing amounts of Dowsil 1700. Figure 5 displays the retention of the water-filled pores after an immediate cure, after 48 h, and after 7 days. All specimens studied longer than an immediate cure were stored at 3 °C to refrain from PDMS curing at ambient temperature. Coalescence of the water molecules occurs just 48 h after it is stored away. After 7 days, the coalescence increased dramatically and resulted in a polydispersed system. The rapid coalescence of water molecules is attributed to low interfacial tension between Sylgard 184 precursor and water [31]. To increase the viscosity of the PDMS and further increase the stability of the water in the oil emulsion system, Dowsil 1700 is added to the Sylgard 184 as described in Table 3. Figure 6 depicts the effects of adding Dowsil 1700, a more viscous PDMS base, on pore size and porosity percentage over time. The incorporation of a high-viscous oil at the same mixing rate resulted in less porous and lower average pore size systems. However, higher concentrations of Dowsil 1700 led to higher pore stability over time. Figure 7 portrays the stability of water emulsion foams with Dowsil 1700 after three weeks.

### 3.4. Exploration of Storage Procedure

**Figure 8:**
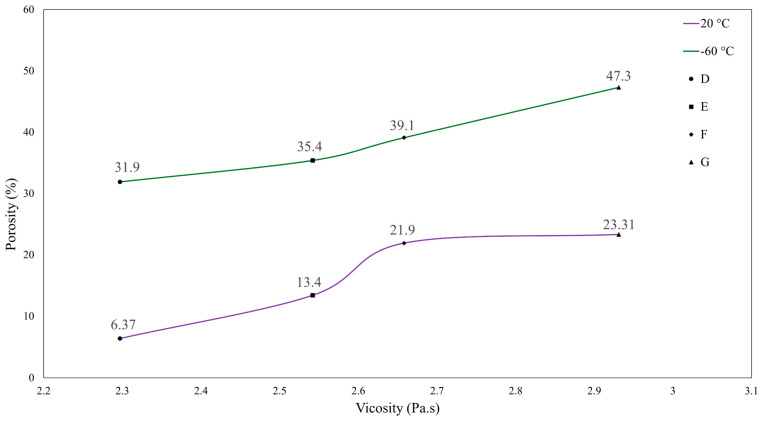
Effects of specimen porosity as a function of specimen precursor viscosity under conditions of −60 °C and 20 °C.

Exploration to promote the retention of the “water-in-oil” emulsion foams was carried out in liquid PDMS samples mixed with water. To achieve stability, the specimens were stored in a freezer at −60 °C for three weeks. This temperature induced the freezing of the water-filled pores, thus restricting the movement of the water molecules that tend to coalesce, as seen before in Figure 5. After freezing, the liquid samples were removed and molded following the same procedure described in Section 2.2. At this point, the specimens have no water in their pores, being fully processed. The freezing mechanism influences the properties of the PDMS matrix by increasing its viscosity [32]. Figure 7B confirms the stability of the water emulsion by upholding a similar average pore size and porosity percentage throughout the foam after three weeks. Figure 8 compares the 3-week storage life of water emulsion foams at different viscosities and different storage conditions. Green data points indicate specimens that were stored for 3 weeks at −60 °C. Purple data points signify specimens that were stored for 3 weeks at 20 °C. The water emulsion foam data points from left to right correspond to specimens D, E, F, and G, as described in Table 3. The optimal pore retention was achieved by specimen G in both storage methods due to a higher matrix viscosity of 2.931 Pa·s. This conclusion is supported by the literature, as high steric stability is achieved in highly viscous systems [33]. This behavior can be attributed to the high viscosity of the matrix slowing down the movement of the particles, ensuring that the pores do not coalesce. Nevertheless, specimens stored in the −60 °C environment displayed superior pore retention effects. The lowest recorded porosity percentage of foams stored at −60 °C was 31.9%, approximately a 37% increase from the maximum recorded porosity for the specimens stored at 20 °C.

### 3.5. Rheological Properties of Water Emulsion Foams

**Figure 9:**
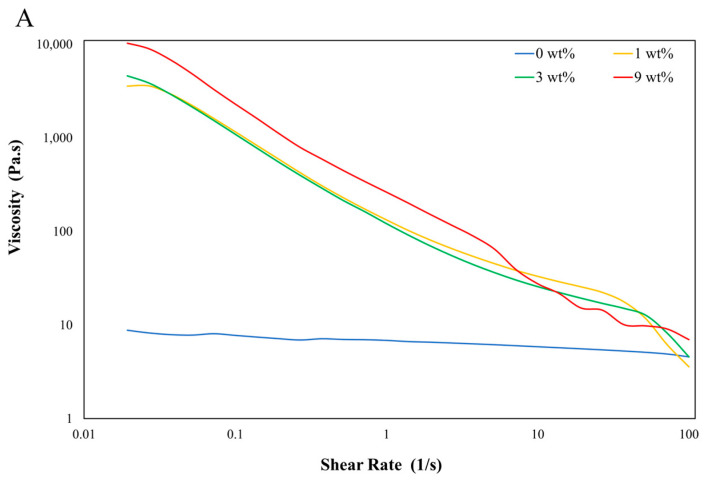

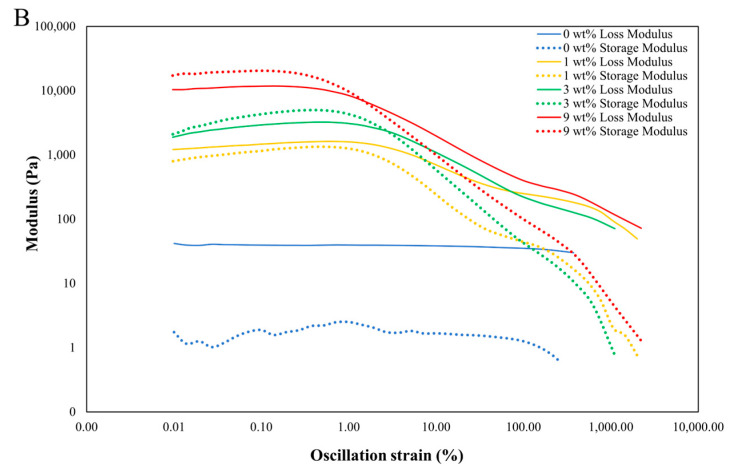
Rheological properties of water emulsion foams show (**A**) shear thinning and (**B**) shape retention behavior.

Successful DIW printing of polymers is dictated by their rheological properties. Inks employed in DIW must have their properties finetuned to achieve thixotropic behavior and shear thinning within the AM system’s parameters. Challenges arise during this process as rheological qualities for correct extrusion are dictated by the mixing ratios of the material blends, sometimes below the micron scale [34]. Balancing rheological properties for extrusion and thixotropy for adequate shape retention is crucial and is the driving factor for successful DIW of viscoelastic materials. In the present study, various amounts of fumed silica were used to modify the ink’s rheology and induce thixotropic behavior, including a control sample with no fumed silica. These formulations may be seen in Table 4. The viscosities of the different ink formulations were plotted against the shear rate, as shown in Figure 9A. This plot illustrates that shear thinning starts occurring at low shear rates for Trials 2, 3, and 4, which contain 1 wt%, 3 wt%, and 9 wt% of fumed silica particles, respectively. On the other hand, Trial 1 (no fumed silica) displays an almost constant viscosity with increasing shear rate, meaning that shear thinning is not taking place and the material is Newtonian. Shear thinning of gel emulsions occurring at room temperature allows the extrusion of these materials using a deposition nozzle with excellent mechanical properties, such as high shear yield strength [35]. With the increase in the applied shear rate, formulations containing fumed silica increased the maximum viscosity experimented by the ink. Table 4 reports the inks’ maximum viscosity value, the highest being Trial 4, which had a maximum viscosity value of 10,290.8 Pa·s. From the viscosity plots, it is evident that fumed silica has a large effect on the ink’s rheological behavior. This is concurrent with research in the literature by Pacquien et al. [36]. Pacquien et al., displayed that, at low volume fractions of rheology modifier, G″ is lower than G′. However, at higher volume fractions, a crossover between G′ and G″ occurs. Lack of shear thinning does not necessarily imply the material cannot be extruded. If viscosity is sufficiently low material can be extruded as it occurred with Trial 1 (0 wt%). However, there is another crucial parameter that governs the ink’s rheology once it has been laid down: thixotropic shape retention.

Materials that exhibit thixotropy do not require much time to go back to a solid state compared to non-thixotropic materials. The storage modulus to loss modulus ratio of the ink is paramount in dictating if 3D structures can self-support several layers after being printed and thus retain their shape. Inks with a storage modulus (G′) lower than the loss modulus (G″) exhibit Newtonian or liquid-like behavior [37]. Due to the liquid-like flow, these types of inks cannot withstand the weight of various layers of printed materials or maintain their extruded shape. Qin et al., pointed out that G′ values higher than 1 kPa are necessary for self-supporting 3D structures with more than two vertical layers [38]. The shear strain was oscillated for three trials containing samples with different fumed silica concentrations and one with no fumed silica particles. Figure 9B shows the moduli against oscillation strain for the four samples. Trial 1 with no fumed silica displays G′ values below 10 Pa and it is always lower than G″, while Trial 2, which shows a shear thinning response in Figure 9A, has similar values for both moduli, but G′ is still lower than G″ across all oscillation strains. This ink can be extruded but shows spreading compared to Trial 3, which contains 3 wt% of fumed silica particles but a different matrix formulation. On the other hand, Trial 3 saw a higher G′ than G″ for the region before the yield point at 4.88%, as shown in Figure 9B, followed by a region with higher G″ after this yield point. This change in the moduli indicates a change between solid-like and liquid-like properties, as the material has now become a viscoelastic liquid. Trial 4, containing the highest fumed silica concentration at 9 wt%, also shows two regions divided by a yield point, with a higher G′ in the low-stress region. Trials 3 and 4 ensure shape retention under low shear stresses as they maintain solid-like properties and possess high-yield shear stress to withstand several layers. The moduli and viscosities increased with rising fumed silica concentrations; each trial required a higher pressure than the one before to extrude. In general, inks for DIW should have G′ higher than G″ as well as high enough G′ values to support the layers’ weights [39]. Trials 3 and 4 exhibit excellent shear thinning and thixotropic shape retention for DIW printing of 3D structures. The adaptable mechanical properties made possible during synthesis and its feasibility for 3D printing grant the opportunity to combine tunable structures with designs for additive manufacturing. Montazerian et al., printed triply periodic minimal surfaces with PDMS, discovering that radially gradient pore distribution increases elastic modulus. In addition to this, Montazerian et al., reported that different designs lead to stiffer parts [40]. The presented work adds to the already exemplary versatility of PDMS through PDMS, resulting in parts that may have great shape retainability with tunable mechanical properties [41].

## 4. Conclusions

In this study, a distinct method was presented for the fabrication of PDMS foams with regulated porosity by adjusting water proportions. Furthermore, optimization of these PDMS foams was achieved through the incorporation of a rheological modifier, specifically for DIW 3D printing. The results demonstrated a direct relationship between increasing water percentage and larger pore diameters. Notably, specimen A, with a PDMS-to-water ratio of 65:35, produced a 30% porous sample with an average pore diameter of 4.22 µm. This particular specimen exhibited superior mechanical properties compared to the other samples, boasting a Young’s Modulus of 1.17 MPa. This is 193%, 60%, and 31.5% higher than specimens B, C, and Z, respectively. The energy absorption of specimen A dwarfs other samples, with a 200% increase from specimen B, a 154% increase from specimen C, and a 120% increase from a bulk sample. Lastly, specimen A also hosted the highest compressive stress, leading to increases of 115%, 185%, and 192% from specimens B, C, and Z, respectively. To extend the shelf life of specimen C, Dowsil 1700 was incorporated. The optimal storage temperature was identified as −60 °C, demonstrating improved pore stability over three weeks. The impact of fumed silica on rheology was investigated, revealing that a 9 wt% concentration of fumed silica yielded rheological data suitable for DIW printing, along with exhibiting shear-thinning and thixotropic behavior. The ability to tune porosity, mechanical properties, and rheology implies a significant and broad impact of this work across diverse industries where PDMS is used frequently. This work stands to benefit the state-of-the-art in medical use, combining tunable mechanical properties with improvements in antimicrobial properties, antibacterial properties, and reducing capsular contracture that leads to implant failure [42,43,44]. The potential of adding self-replenishing hydrophobic coatings to this tunable material is notable to the space industry, especially considering the current resistance of these materials in a low earth orbit environment [45,46]. The defense industry may utilize the adaptable nature of this work by incorporating flexibility with PDMS-Ni coated composites used for microwave absorption, resulting in a reduced rate of detection for vehicles or protecting small, sensitive equipment from electromagnetic influence [47].

## Figures and Tables

**Table 1:** Porosity percentages and average pore sizes of water emulsion foams at varying PDMS-to-water ratios.

Specimen	PDMS/Water Ratio (wt%)	Average Pore Diameter (µm)	Average Porosity (%)
Z (Bulk)	100:0	0.00	0.00
A	65:35	4.22	30.2
B	35:65	4.91	55.5
C	10:90	5.35	51.9

**Table 2:** Mechanical properties of water emulsion foams.

Specimen	Modulus of Elasticity (MPa)	Energy Absorption (J/mm^3^)	Compressive Strength (MPa)
Z (Bulk)	0.89	0.15	1.20
A	1.17	0.33	3.50
B	0.40	0.11	1.63
C	0.73	0.13	1.23

**Table 3:** Formulations of water emulsion foams using Sylgard 184 and Dowsil and their respective viscosities.

Specimen	Sylgard 184:Dowsil 1700 (wt%)	PDMS/Water (wt%)	Average Pore Diameter (µm)	Porosity (%)	Experimental Viscosity (Pa·s)
A	100:0	65:35	6.48	30.2	2.013
D	90:10	65:35	5.47	20.4	2.297
E	80:20	65:35	4.22	18.2	2.542
F	70:30	65:35	2.86	9.4	2.658
G	60:40	65:35	2.01	9.2	2.931

**Table 4:** Water emulsion ink compositions optimized for DIW printing.

Trial #	Sylgard 184:Dowsil 1700 (wt%)	PDMS/Water (wt%)	Fumed Silica Particles (wt%)	Experimental Viscosity (Pa·s)
1	30:70	65:35	0	8.8
2	50:50	65:35	1	3583.1
3	40:60	65:35	3	4612.7
4	70:30	65:35	9	10,290.8

## Data Availability

Data are contained within the article.
